# Effects of denosumab on bone mineral density and bone turnover markers in rheumatoid arthritis patients switching from bisphosphonates

**DOI:** 10.1186/s40634-019-0211-7

**Published:** 2019-10-29

**Authors:** Tetsuya Kaneko, Koichi Okamura, Yukio Yonemoto, Chisa Okura, Takahito Suto, Masahiro Tachibana, Hideo Sakane, Makoto Inoue, Hirotaka Chikuda

**Affiliations:** 1 Department of Orthopaedic Surgery, Japan Redcross Society Fukaya Red Cross Hospital, Fukaya, Saitama, Japan; 2 Department of Orthopaedic Surgery, Gunma University Graduate School of Medicine, 3-39-22 Showamachi, Maebashi, Gunma Japan; 3 Department of Orthopaedic Surgery and Rheumatology, Inoue Hospital, Takasaki, Gunma Japan; 4 Department of Orthopaedic Surgery, Fukaya Seikeigeka Clinic, Fukaya, Saitama, Japan

**Keywords:** Osteoporosis, Denosumab, Bisphosphonate, Rheumatoid arthritis, Fragility fractures

## Abstract

**Background:**

To compare the efficacy of 12-month denosumab treatment on bone mineral density (BMD) and bone turnover markers (BTMs) between treatment-naïve osteoporosis patients with rheumatoid arthritis (RA) and those with previous bisphosphonate (BP) therapy.

**Methods:**

A total of 36 RA patients with osteoporosis completed 12-month follow-up. Twenty-five patients were osteoporotic treatment-naïve (naïve group), and 11 patients were previously treated with BPs (switch group) (average 7.9 years). BMD and BTMs were measured before and 6 and 12 months after treatment.

**Results:**

BTM levels were higher in the naïve group at baseline. However, the same level of suppression was achieved at 6 months in both groups. Spine BMD increased significantly in both groups. There was no significant difference in the mean percent changes of BMD of the spine (naïve group: 6.8 ± 0.8, switch group: 5.1 ± 1.5), femoral neck (2.9 ± 1.4, 2.9 ± 1.3), and total hip (1.7 ± 0.9, 1.4 ± 1.1) between these two groups at 12 months.

**Conclusions:**

The effects of denosumab on BMD and BTMs of the switch group after long-term BP treatment are comparable to those of the naïve group in RA patients. Thus, switching BPs to denosumab is one of the useful options to treat osteoporosis with RA.

## Background

Rheumatoid arthritis (RA) is an inflammatory disease in which systemic arthritis causes significant functional impairment. Generalized bone loss leading to osteoporosis is one of the extra-articular manifestations of RA (Vis et al. 2006) and may lead to the occurrence of fragility fractures, which further increase the burden of the disease. Therefore, RA patients often require treatment for osteoporosis in addition to RA treatment. RA-related risk factors for osteoporosis, such as body mass index (BMI), disease duration and activity, level of disability, corticosteroid and disease-modifying anti-rheumatic drug (DMARD) usage, menopausal status, and production of pro-inflammatory cytokines, have been classified and studied (Gough et al. 1994; Haugeberg et al. 2000; Laan et al. 1992; Sinigaglia et al. 2000). It is known that upregulation of pro-inflammatory cytokines such as tumor necrosis factor (TNF)-α, interleukin (IL)-1, and IL-6 leads to bone resorption through overexpression of receptor activator of nuclear factor kappa-B ligand (RANKL) and activated T cells (Kotake et al. 2001).

Anti-resorptive agents, such as bisphosphonates (BPs), have a high affinity for bone minerals and inhibit bone resorption by osteoclasts (Reszka and Rodan 2003). BPs have high-level evidence for fracture risk reduction in postmenopausal osteoporotic women (Wells et al. 2008a; Wells et al. 2008b). Some reports have shown that several BPs (alendronate, risedronate, zoledronate) have positive effects on bone mineral density (BMD) in patients with inflammatory rheumatic conditions (Benucci et al. 2009; den Uyl et al. 2011; Hakala et al. 2012). Therefore, BPs have mainly been used for the prevention of fragility fractures in RA patients. However, there is no evidence for how long osteoporotic patients should be treated with BPs, and which agent should be administered to RA patients with osteoporosis after the treatment with BPs.

Another anti-resorptive agent, denosumab, is an antibody against RANKL, which is essential for osteoclast differentiation, activation, and survival (Fuller et al. 1998; Lacey et al. 2000; Lacey et al. 1998; Yasuda et al. 1998). Denosumab potently reduces bone resorption through binding RANKL, leading to increases in BMD and cortical bone microstructure in postmenopausal patients with osteoporosis (Genant et al. 2010; Poole et al. 2015). Moreover, previous reports of denosumab have shown not only increases in lumbar spine and hip BMD, but also inhibition of progression of bone erosion in RA patients (Cohen et al. 2008; Mok et al. 2015; Rossini et al. 2016; Takeuchi et al. 2016). These observations suggest that denosumab has positive effects on not only the prevention of fragility fractures, but also on joint damage in RA patients.

Recently, Ebina et al. reported that the switching BPs to denosumab showed a significant increase in BMD compared to those with continued BPs in RA patients, and concluded that switching BPs to denosumab in RA might provide some useful osteoporosis treatment options (Ebina et al. 2018). Therefore, it should be evaluated whether the clinical efficacy of denosumab following BPs could be comparable to those with treatment-naïve patients with osteoporosis in RA.

The purpose of this study was to compare the effects of denosumab on BMD and bone remodeling markers between treatment-naïve patients and patients previously treated with long-term BPs.

## Materials and methods

### Patients

This study was an observational study in the Gunma Rheumatoid Arthritis Network, and the ethics committee of Gunma University approved the protocol (IRB approval No 23–37). Informed consent was obtained from all individual participants included in the study. The subjects were 42 women RA patients aged 50 years and older who started receiving denosumab treatment from May 2014 to June 2015 at our hospital. All patients fulfilled the American College of Rheumatology (ACR) classification criteria (1987) (Arnett et al. 1988) and they had received anti-rheumatic drugs (glucocorticoids (PSL), salazosulfapyridine, iguratimod, bucillamine, tacrolimus, methotrexate (MTX), bailogical disease-modifying anti-rheumatic drugs (bDMARDs)) prior to this study. The patients were diagnosed with concurrent osteoporosis, defined as 1) femoral neck or vertebral fragility fractures or 2) young adult mean BMD less than 70% without a fragility fracture. Patients with other inflammatory diseases, class 4 Steinbrocker classification (Steinbrocker et al. 1949), serious renal or hepatic dysfunction, or previous spinal or hip procedures were excluded. Twenty-eight of 42 patients were treatment-naïve osteoporotic RA patients. The effects of denosumab administered to RA patients with no previous treatment (naïve group) were compared to those with BP pre-treatment (switch group). Denosumab (60 mg, Daiichi-Sankyo, Tokyo, Japan) was injected subcutaneously every 6 months. All patients received supplements of calcium 610 mg, magnesium 30 mg and vitamin D 400 IU per day throughout the study. The mean duration of prior BP treatment was 7.9 years in the switch group. The BPs that had been used were alendronate (*n* = 3) and risedronate (*n* = 8).

### Clinical assessment

The serum levels of total-procollagen type I N-terminal propeptide (total-PINP), tartrate-resistant acid phosphatase-5b (TRACP-5b), and undercarboxylated osteocalcin (ucOC), and the BMDs of the lumbar spine (LS) (L1-L4), the left femoral neck (FN), and the left total hip (TH) on dual-energy X-ray absorptiometry (DCS-900EX; Hitachi, Tokyo, Japan) were measured before and 6 and 12 months after the start of treatment. The disease activity scores of 28 joints based on the erythrocyte sedimentation rate (DAS28-ESR) and the simplified disease activity index (SDAI) were used to evaluate RA disease activity (Aletaha and Smolen 2005). Use of methotrexate (MTX), glucocorticoids (PSL), biological disease-modifying anti-rheumatic drugs (bDMARDs), and other conventional synthetic disease-modifying anti-rheumatic drugs (csDMARDs) was also investigated.

### Statistical analysis

Statistical analysis was conducted using SPSS version 22 (IBM Inc., Chicago, IL, USA). Results are reported as means ± standard deviation (SD). A *p* value less than 0.05 was considered significant. Chi-squared tests or Fisher’s exact tests were used for comparisons between two groups for categorical variables, and Mann-Whitney *U* tests were used to assess continuous variables. Wilcoxon’s signed-rank sum test or McNemar’s test was used to assess differences between the groups, as appropriate.

## Results

**Fig. 1:**
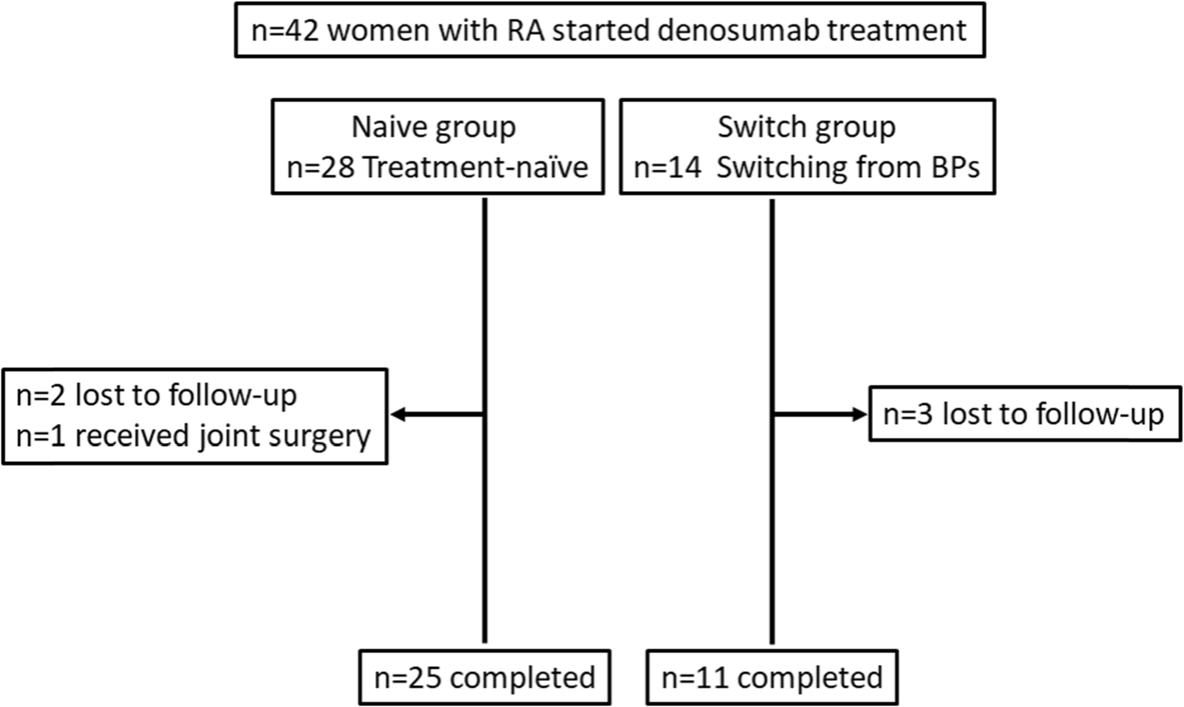
Trial profile. RA: rheumatoid arthritis; Naive group: treatment-naïve group; Switch group: transitioning from bisphosphonate group; BPs: bisphosphonates

A total of 36 patients completed 12-month follow-up. A total of 6 patients discontinued the study, 3 in the naïve group and 3 in the switch group; of these patients, 2 in the naïve group and 3 in the switch group discontinued because they were lost to follow-up, and 1 patient in the naïve group discontinued because of joint surgery (Fig. 1).

The clinical characteristics of the patients are shown in Table 1. There were no significant differences in height, weight, and BMD between the groups. However, mean age and percentage with previous fragility fractures were significantly higher in the switch group than in the naïve group. Furthermore, baseline total-P1NP, TRACP-5b, and ucOC levels were significantly higher in the naïve group than in the switch group.

**Table 1:** Baseline characteristics

Baseline characteristic	Naïve group (*n* = 25)	Switch group (*n* = 11)	*P*
Age (years)	66.2 ± 1.6	75.4 ± 2.2	<0.05
Height (cm)	150.4 ± 1.5	145.4 ± 2.5	n.s
Weight (kg)	45.7 ± 1.6	43.0 ± 1.9	n.s
Spine BMD (g/cm^2^)	0.824 ± 0.03	0.889 ± 0.06	n.s
FN BMD (g/cm^2^)	0.60 ± 0.01	0.57 ± 0.03	n.s
TH BMD (g/cm^2^)	0.62 ± 0.01	0.62 ± 0.03	n.s
Total-PINP (ng/ml)	73.4 ± 5.5	36.8 ± 5.1	n.s
TRACP-5b (mU/dl)	426.2 ± 31.3	320.7 ± 21.0	<0.05
ucOC (ng/ml)	4.2 ± 0.5	2.3 ± 0.4	<0.05
BPs duration (years)	–	7.9 ± 0.4	–
BPs used	–	Alen 3, Ris 8	–
Previous fractures (%) ^a^	28.0	81.8	<0.05

Naïve group: treatment-naïve group; Switch group: transitioning from bisphosphonate group; BMD: bone mineral density; FN: femoral neck; TH: total hip; total-PINP: total-procollagen type I N-terminal propeptide; TRACP-5b: tartrate-resistant acid phosphatase-5b; ucOC: undercarboxylated osteocalcin; BPs: bisphosphonates; Alen: alendronate; Ris: risedronate

^a^Defined as osteoporotic vertebral or nonvertebral fractures; assessment of vertebral fractures was performed for this study at baseline

Data are presented as means ± standard deviation (SD). *P* values from Mann-Whitney *U* tests for continuous variables and Chi-squared tests for dichotomous variables

RA disease duration, disease activity, and medical condition at baseline were not significantly different between the 2 groups (Table 2).

**Table 2:** Baseline characteristics of RA

Baseline characteristic	Naïve group (*n* = 25)	Switch group (*n* = 11)	*P*
Disease duration (years)	13.2 ± 2.0	15.5 ± 2.7	n.s
Steinbrocker			
Stage (I/II/III/IV)	1/2/4/18	0/2/1/7	n.s
Class (1/2/3/4)	2/8/15/0	0/3/8/0	n.s
RF positive (%)	68.0	72.7	n.s
ACPA positive (%)	72.0	81.8	n.s
DAS28-ESR	3.60 ± 0.17	4.15 ± 0.32	n.s
SDAI	6.90 ± 1.00	9.93 ± 1.38	n.s
MTX (%)	60.0	45.5	n.s
MTX dose (mg/week)	8.1 ± 0.9	7.2 ± 1.4	n.s
PSL (%)	32.0	54.5	n.s
PSL dose (mg/day)	5.3 ± 1.8	3.2 ± 0.6	n.s
bDMARDs (%)	32.0	54.5	n.s
bDMARDs	IFX 2, ADA 1, TCZ 3, GLM 1, ABT 1	ETN 2, TCZ 1, GLM 2, ABT 1	
csDMARDs (%)	36.0	27.3	n.s

Data are presented as means ± standard deviation (SD), RA: rheumatoid arthritis; Naïve group: treatment-naïve group; Switch group: transitioning from bisphosphonate group; ACPA: anti-cyclic citrullinated peptide antibody; RF: rheumatoid factor; DAS: disease activity score; SDAI: simplified disease activity score; MTX: methotrexate; PSL: prednisolone; bDMARDs: biological disease-modifying anti-rheumatic drugs; csDMARDs: conventional synthetic disease-modifying anti-rheumatic drugs (salazosulfapyridine, iguratimod, bucillamine, tacrolimus); TCZ: tocilizumab; ETN: etanercept; ADA: adalimumab; ABT: abatacept

*P* values from Mann-Whitney *U* tests for continuous variables and Chi-squared tests or Fisher’s exact tests for dichotomous variables

Table 3 shows the changes in DAS28-ESR, SDAI, and the use of MTX, PSL, bDMARDs, and csDMARDs during this study. These parameters also showed no significant differences from baseline to 6 and 12 months after treatment initiation.

**Table 3:** Changes of disease activities and medications

	Naïve group (*n* = 25)			Switch group (*n* = 11)		
Variable	Baseline	6 months	12 months	Baseline	6 months	12 months
DAS28-ESR	3.60 ± 0.17	3.55 ± 0.14	3.57 ± 0.16	4.15 ± 0.32	4.07 ± 0.32	3.93 ± 0.30
SDAI	6.90 ± 1.00	7.34 ± 0.99	7.36 ± 0.99	9.93 ± 1.38	10.1 ± 2.00	9.52 ± 2.09
MTX (%)	60.0	60.0	60.0	45.5	45.5	45.5
MTX dose (mg/week)	8.1 ± 0.9	7.9 ± 1.0	7.7 ± 1.0	7.2 ± 1.4	7.2 ± 1.4	7.2 ± 1.4
PSL (%)	32.0	28.0	32.0	54.5	54.5	63.6
PSL dose (mg/day)	5.3 ± 1.4	4.7 ± 1.0	4.0 ± 0.9	3.2 ± 0.6	3.2 ± 0.6	3.4 ± 0.6
bDMARDs (%)	32.0	32.0	32.0	54.5	54.5	54.5
csDMARDs (%)	36.0	40.0	40.0	27.3	27.3	27.3

Data are presented as means ± standard deviation (SD), Naïve group: treatment-naïve group; Switch group: transitioning from bisphosphonate group; DAS: disease activity score; SDAI: simplified disease activity score; MTX: methotrexate; PSL: prednisolone; bDMARDs: biological disease-modifying anti-rheumatic drugs; csDMARDs: conventional synthetic disease-modifying anti-rheumatic drugs (salazosulfapyridine, iguratimod, bucillamine, tacrolimus); *P* values from Wilcoxon’s rank sum test for continuous variables and McNemar’s test for dichotomous variables for the change from baseline within each treatment group**.** There was no significant change from baseline in all parameters

Table 4 shows the changes of serum levels of total-PINP, TRACP-5b, and ucOC and of BMD. In the naïve group, there were significant decreases in all BTMs at 6 months and 12 months from baseline. On the other hand, in the switch group, there were no significant decreases in all BTMs at 6 and 12 months compared to baseline except for ucOC at 6 months. At 6 and 12 months, the BTM levels were not significantly different between the two groups, although baseline levels were higher in the naïve group than in the switch group (Table 5). Spine BMD was significantly increased in both groups from baseline to 6 and 12 months. Femoral neck (FN) BMDs at 6 months in both groups and at 12 months in the naïve group were significantly increased from baseline. However, in the switch group, there was no significant increase in FN BMD at 12 months. TH BMD was significantly increased only in the naïve group at 12 months.

**Table 4:** Changes of bone turnover markers and BMD in the naïve and switch groups after 6 and 12 months of treatment

	Naïve group (*n* = 25)					Switch group (*n* = 11)				
	Baseline	6 months	*P*	12 months	*P*	Baseline	6 months	*P*	12 months	*P*
Total-PINP (ng/ml)	73.4 ± 5.5	30.6 ± 3.6	<0.05	32.5 ± 3.5	<0.05	36.8 ± 5.1	25.6 ± 3.8	n.s	29.9 ± 5.7	n.s
TRACP-5b (mU/dl)	426.2 ± 31.3	244.8 ± 27.6	<0.05	250.2 ± 22.4	<0.05	320.7 ± 21.0	278.4 ± 47.9	n.s	276.6 ± 40.0	n.s
ucOC (ng/ml)	4.1 ± 0.5	1.6 ± 0.2	<0.05	1.5 ± 0.2	<0.05	2.3 ± 0.6	1.1 ± 0.3	<0.05	1.4 ± 0.4	n.s
Spine BMD (g/cm^2^)	0.83 ± 0.04	0.88 ± 0.05	<0.05	0.89 ± 0.05	<0.05	0.85 ± 0.04	0.88 ± 0.04	<0.05	0.89 ± 0.04	<0.05
FN BMD (g/cm^2^)	0.60 ± 0.01	0.62 ± 0.01	<0.05	0.62 ± 0.01	<0.05	0.57 ± 0.03	0.59 ± 0.03	<0.05	0.59 ± 0.02	n.s
TH BMD (g/cm^2^)	0.62 ± 0.01	0.63 ± 0.02	n.s	0.64 ± 0.02	<0.05	0.62 ± 0.03	0.63 ± 0.03	n.s	0.63 ± 0.03	n.s

Data are presented as means ± standard deviation (SD), Naïve group: treatment-naïve group; Switch group: transitioning from bisphosphonate group; BMD: bone mineral density; total-PINP: total-procollagen type I N-terminal propeptide; TRACP-5b: tartrate-resistant acid phosphatase-5b; ucOC: undercarboxylated osteocalcin; FN: femoral neck; TH: total hip. *P* values from Wilcoxon’s rank sum test for the change from baseline within each treatment group

**Table 5:** The bone turnover markers levels at 6 and 12 months in the naïve and switch groups

	6 months			12 months		
	Naïve group (*n* = 25)	Switch group (*n* = 11)	*P*	Naïve group (*n* = 25)	Switch group (*n* = 11)	*P*
Total-PINP (ng/ml)	30.6 ± 3.6	25.6 ± 3.8	n.s	32.5 ± 3.5	29.9 ± 5.7	n.s
TRACP-5b (mU/dl)	244.8 ± 27.6	278.4 ± 47.9	n.s	250.2 ± 22.4	276.6 ± 40.0	n.s
ucOC (ng/ml)	1.6 ± 0.2	1.1 ± 0.3	n.s	1.5 ± 0.2	1.4 ± 0.4	n.s

Data are presented as means ± standard deviation (SD), Naïve group: treatment-naïve group; Switch group: transitioning from bisphosphonate group; total-PINP: total-procollagen type I N-terminal propeptide; TRACP-5b: tartrate-resistant acid phosphatase-5b; ucOC: undercarboxylated osteocalcin. *P* values from Mann-Whitney *U* tests, for the bone turnover markers in the naïve group versus the switch group

**Fig. 2:**
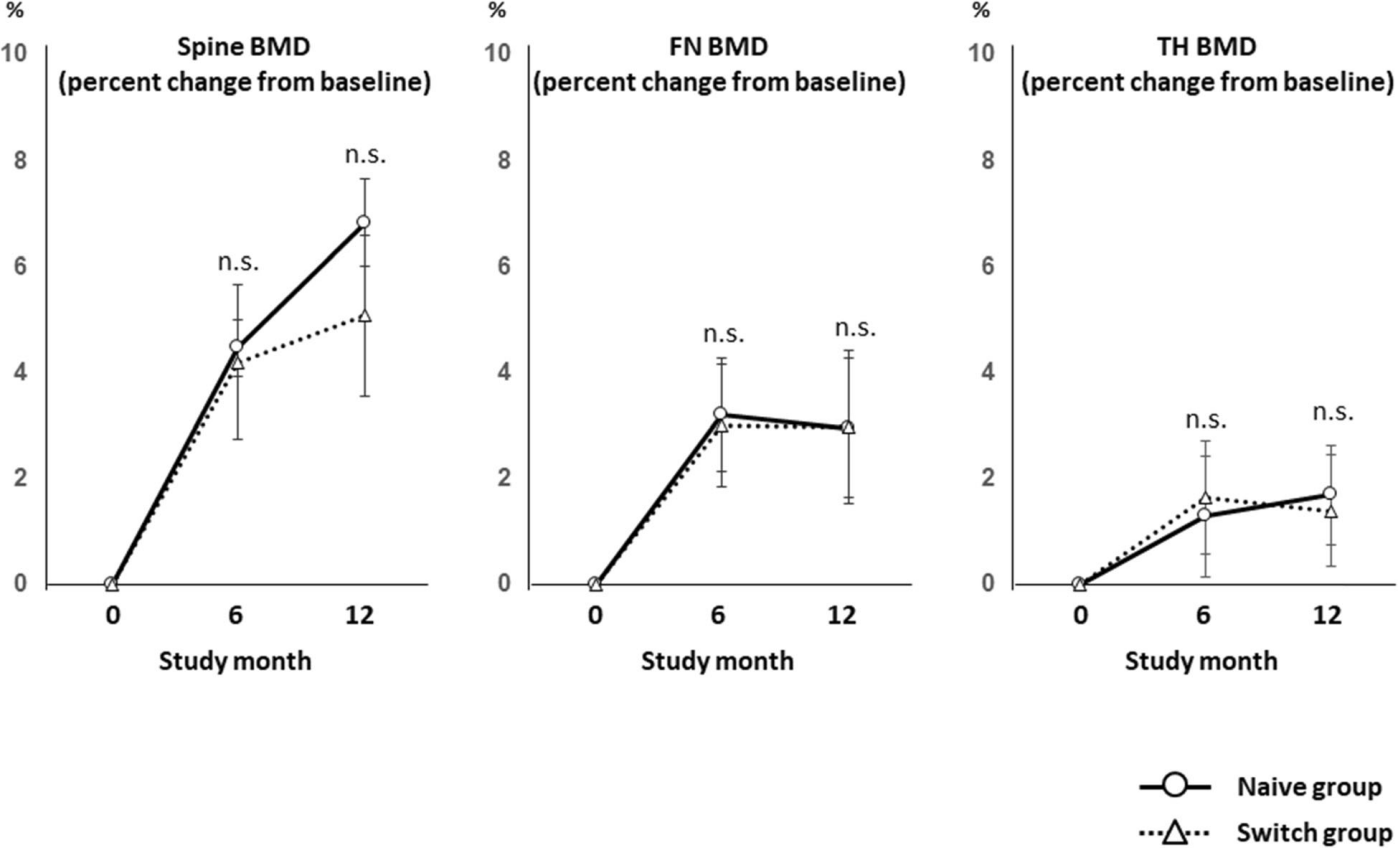
Mean percent changes from baseline in lumbar spine and hip BMDs. Naive group: treatment-naïve group; switch group: transitioning from bisphosphonate group; BMD: bone mineral density; FN: femoral neck; TH: total hip. *P* values from Mann-Whitney *U* tests, for the mean percent change from baseline in the naive group versus the switch group. n.s denotes no significant difference between the values

Figure 2 shows the mean percent changes of lumbar spine and hip BMDs from baseline. There were no significant differences between the groups in the mean percent changes of BMD of the spine, FN, and TH at 6 months (naïve group: 4.5 ± 0.5, 3.2 ± 1.1, 1.3 ± 1.1, switch group: 4.2 ± 1.4, 3.0 ± 1.2, 1.6 ± 1.1, respectively) and at 12 months (naïve group: 6.8 ± 0.8, 2.9 ± 1.4, 1.7 ± 0.9, switch group: 5.1 ± 1.5, 2.9 ± 1.3, 1.4 ± 1.1, respectively). No subjects experienced any clinical fractures or serious adverse effects during the course of this study.

## Discussion

In this study, the effect of denosumab in RA patients previously treated with long-term (average 7.9 years) BPs (switch group) was compared to that in treatment-naïve RA patients (naïve group). At 6 and 12 months, the BTM levels were similar between the two groups, although baseline levels in the naïve group were significantly higher than those in the switch group.

Nakamura et al. reported a direct comparison of BMD changes between the treatment-naïve and the BPs pre-treated patients at the 24 months. They demonstrated that the denosumab had an equal effect on the BMD increase to the treatment-naïve group and the group with BPs treatment over 15 years (Nakamura et al. 2017). Although the duration of pre-treatment with BPs was shorter than their study, this study showed significantly increased spine BMD in both groups from baseline to 6 and 12 months, and the significant increase in FN BMD was observed from baseline to 6 months in both groups. In addition, the mean percent changes in BMD were not significantly different between the two groups.

A similar study of denosumab for postmenopausal patients reported a direct comparison of BMD changes at 12 months in treatment-naïve patients and patients pre-treated with zoledronic acid. Pre-treated postmenopausal patients showed significant increases in lumbar spine and hip BMDs, and these increases did not differ significantly between the two groups (Anastasilakis et al. 2015). Moreover, previous reports have indicated that BPs reduce bone resorption through a different mechanism than denosumab (Lacey et al. 1998; Russell et al. 2008). The present results also suggest that prior BP treatment does not negatively affect subsequent denosumab treatment in RA patients, at least in the short term.

On the other hand, in the switch group, there was no significant increase in FN and TH BMDs from baseline to 12 months. Moreover, the mean percent changes of lumbar BMD were larger in the naïve group than in the switch group, although the difference was not significant (Fig. 2). The previous study also reported that denosumab without pre-treatment had a significantly higher percent change in the hip total BMD at 6 months in RA patients (Nakamura et al. 2017). These might be attributable to the already-decreased bone remodeling due to BP treatment before initiation of denosumab treatment.

In this study, BTM levels were higher in the naïve group at baseline as expected. In the naïve group, they were significantly decreased from baseline at all time periods. In the switch group, they were not significantly decreased, except for ucOC at 6 months. However, although starting from a much different baseline level, BTMs reached similar levels of suppression at 6 months in both groups (Table 5). This finding suggests that previous treatment with BPs, such as alendronate or risedronate, does not attenuate the suppression of bone turnover achieved by denosumab.

Inhibition of RANKL has been shown to prevent inflammatory bone loss in patients with chronic inflammatory arthritis (Schett et al. 2005) and to prevent both bone erosion in the subchondral bone and cartilage destruction (Takeuchi et al. 2016). Since this study demonstrated that short term administration of denosumab had a significant increase in BMD and decrease in the BTM levels, the denosumab could be the first osteoporotic agent for the treatment with osteoporosis in RA patients. On the other hand, in the present study, denosumab did not affect disease activity, including inflammation, in RA patients, similar to the findings of a past report (Kinoshita et al. 2016). From these observations, we consider that when using denosumab to treat RA, it is better to use it in combination with some anti-rheumatic disease agents.

In general, there is a positive relationship between the course of RA and the degree of both local and generalized bone losses, that is, cumulatively increased disease activity is associated with systemic bone loss. Therefore, treatment with DMARDs is of major importance, not only to improve disease activity, but also to prevent generalized bone loss. Several studies have shown that bDMARD treatment, mostly combined with MTX, protected against generalized bone loss and impaired bone quality (Kageyama et al. 2008; Marotte et al. 2007; Wijbrandts et al. 2009). Moreover, the use of glucocorticoids increases the risk of fragility fracture due to inhibition of the production, survival, and differentiation of osteoblasts (Chua et al. 2003; Compston 2010; Emkey and Epstein 2014) and enhancement of the survival and activity of osteoclasts by increasing RANKL expression (Sivagurunathan et al. 2005). In the present study, the percentage using PSL was higher in the switch group than in the naïve group. This background difference between the groups may have had a slight effect on the results.

All subjects of this study had received some kind of DMARD treatment for RA, and the percentage of patients using bDMARDs was 32.0% in the naïve group and 54.5% in the switch group. Although there was slightly higher, but not significantly higher, use of bDMARDs in the switch group, use of bDMARDs was considered to have little effect on the changes in BMD and BTMs, because there was no change of bDMARDs during the 12 months (Table 3). Moreover, previous reports demonstrated that the use of bDMARDs and disease control by bDMARDs had not increased, but decreases in the BMD of the hip, spine, and/or hand were prevented. (Bertoldi et al. 2013; Marotte et al. 2007; Wijbrandts et al. 2009).

It has been reported that oral corticosteroid treatment with more than 5 mg daily of prednisolone or equivalent reduces bone mineral density and causes a rapid increase in the risk of fracture during the treatment period (Van Staa et al. 2002). Previous denosumab studies showed that the increases of BMD in glucocorticoid users were expected to be lower than those reported in studies of postmenopausal women (Brown et al. 2009; Kendler et al. 2010; Mok et al. 2015; Recknor et al. 2013). In the present study, 54.5% of patients in the switch group had used PSL, with an average dosage of 3.2 mg/day. This group also had slightly higher disease activities. Thus, although the dose of PSL did not exceed 5 mg/day, it was possible that the combination of PSL and slightly higher disease activity might have affected the increase in BMD in the switch group.

There are several limitations to this study. First, the number of subjects for each parameter at follow-up was small, and comparisons of these data may thus have been affected by number bias, especially the data for age, previous fragility fractures, and the percentages of use of bDMARDs and glucocorticoids as stated above. Although there was no significant difference between the groups in the percentage of use of bDMARDs and PSL on statistical analysis, the switch group had a significantly older age and a higher percentage of fragility fractures; thus, some conditional bias might be expected in comparisons between these groups. Second, this study was not a randomized, controlled trial; thus, selection bias may have been present. Third, the observation period of 12 months was short, and long-term efficacy remains unknown. Fourth, there was no control group. One was the treatment-naïve patients receiving a placebo, and the other was continued BPs group, which allowed to compare the clinical effect of denosumab over BPs. Finally, fracture risk reductions in these patients were not shown in this study. In order to elucidate the above points, results from a long-term study with a large number of patients would be needed.

## Conclusions

In conclusion, a direct comparison between the switch group from long-term BPs treatment and the treatment-naïve group in RA patients demonstrated that there were no significant differences between the groups in the mean percent changes of BMDs of the lumbar spine, FN, and TH at 6 months and 12 months. These data indicated that denosumab’s effectiveness on BMD in the switch group might be comparable to those in the naïve group. Thus, switching BPs to denosumab might be a useful option to treat osteoporosis with RA.

## Data Availability

The data that support the findings of this study are available from the corresponding author upon reasonable request.

## Abbreviations

ACR: American College of Rheumatology
bDMARDs: biological disease-modifying anti-rheumatic drugs
BMD: bone mineral density
BMI: body mass index
BP: bisphosphonate
BTM: bone turnover marker
csDMARDs: conventional synthetic disease-modifying anti-rheumatic drugs
DAS28-ESR: disease activity scores of 28 joints based on the erythrocyte sedimentation rate
DMARD: disease-modifying anti-rheumatic drug
FN: femoral neck
IL: interleukin
MTX: methotrexate
PINP: procollagen type I N-terminal propeptide
PSL: methotrexate
RA: rheumatoid arthritis
RANKL: receptor activator of nuclear factor kappa-B ligand
SD: standard deviation
SDAI: simplified disease activity index
TH: total hip
TNF: tumor necrosis factor
TRACP-5b: tartrate-resistant acid phosphatase-5b
ucOC: undercarboxylated osteocalcin
